# Dysfunction of the Auditory Brainstem as a Neurophysiology Subtype of Autism Spectrum Disorder

**DOI:** 10.3389/fnins.2021.637079

**Published:** 2021-03-17

**Authors:** Jierong Chen, Zhen Wei, Chun Liang, Binguang Liu, Jimin Guo, Xuejun Kong, Minshi Huang, Ziwen Peng, Guobin Wan

**Affiliations:** ^1^Department of Child Psychiatry and Rehabilitation, Affiliated Shenzhen Maternity and Child Healthcare Hospital, Southern Medical University, Shenzhen, China; ^2^Department of Radiology, Affiliated Shenzhen Maternity and Child Healthcare Hospital, Southern Medical University, Shenzhen, China; ^3^Martinos Center, Massachusetts General Hospital, Harvard Medical School, Charlestown, MA, United States; ^4^Center for Studies of Psychological Application, School of Psychology, South China Normal University, Guangzhou, China

**Keywords:** autism spectrum disorder, subtype, speech-ABR, neuroimaging, mediation, neurophysiology

## Abstract

Autism spectrum disorder (ASD) is very heterogeneous, particularly in language. Studies have suggested that language impairment is linked to auditory-brainstem dysfunction in ASD. However, not all ASD children have these deficits, which suggests potential subtypes of ASD. We classified ASD children into two subtypes according to their speech-evoked auditory brainstem response (speech-ABR) and explored the neural substrates for possible subtypes. Twenty-nine children with ASD and 25 typically developing (TD) peers were enrolled to undergo speech-ABR testing and structural magnetic resonance imaging (sMRI). There were significant differences between the ASD group and TD group in surface area, cortical volume and cortical thickness. According to speech-ABR results, ASD participants were divided into the ASD-typical (ASD-T) group and ASD-atypical (ASD-A) group. Compared with the ASD-T group, the ASD-A group had a lower score in language of the Gesell Developmental Diagnosis Scale (GDDS), increased left rostral middle frontal gyrus (lRMFG) area and decreased local gyrification index of the right superior temporal gyrus. GDDS-language and surface area of lRMFG were correlated to the wave-A amplitude in ASD. Surface area of lRMFG had an indirect effect on language performance *via* alteration of the wave-V amplitude. Thus, cortical deficits may impair language ability in children with ASD by causing subcortical dysfunction at preschool age. These evidences support dysfunction of the auditory brainstem as a potential subtype of ASD. Besides, this subtype-based method may be useful for various clinical applications.

## Introduction

Autism spectrum disorder (ASD) is a neurodevelopmental disorder involving deficits in social communication and restricted and repetitive behaviors, with an onset prior to 3 years of age (American Psychiatric Association, 2013). The heterogeneity of individuals with ASD in clinical presentations poses a significant challenge for the interpretability and replicability of research studies (Grzadzinski et al., 2013). Identifying potential subtypes may provide insight into the pathogenesis and/or neurologic mechanism of ASD.

Over the past several decades, researchers have made great efforts to find ways to categorize ASD heterogeneity (Grzadzinski et al., 2013). Classifying individuals with ASD into different groups based on clinical symptoms is one of most widely used methods (Heaton et al., 2008; Deboth and Reynolds, 2017). Besides, numerous studies have used clustering analyses to distinguish different subtypes based on cognitive-behavior and neuropsychology characteristics (Bitsika et al., 2008; Sacco et al., 2012). However, attempts to assign subtypes to individuals with ASD have been largely unsuccessful because distinct, empirically defined subgroups have yet to be identified reliably.

There is an absence of neurobiological evidence for why individuals with ASD present symptoms of varying severity. However, paying attention to the phenotypic behavioral heterogeneity of language in early development is important (Anderson et al., 2007; Pickles et al., 2014). One important reason is that, even though the language trajectories of ASD cases in the first years of life are highly unstable, these trajectories in early childhood are relatively stable and predictive of long-term outcome (Lombardo et al., 2015). Numerous neuroimaging studies have found that abnormalities in the cortex and/or subcortex may be neural mechanisms causing language deficits in ASD (Russo et al., 2008; De Fossé et al., 2010). Nevertheless, the degree of language impairment of individuals with ASD is vastly heterogeneous (Kjelgaard and Tager-Flusberg, 2001). Thus, better understanding of the language-related subtype of ASD in early childhood may help to reveal the neural mechanism that underlies language impairment in such children.

The auditory brainstem response (ABR) is a far-field electric potential measured from the scalp reflecting the electrophysiological activity of the subcortical auditory pathway from the distal portion of the auditory nerve to higher midbrain structures (Skoe et al., 2015). The speech-evoked auditory brainstem response (speech-ABR) is a response to complex sound, and can provide cues as to how temporal and spectral features are preserved in the brainstem (Anderson et al., 2010). Compared with use of questionnaires, the speech-ABR (as an electrophysiological indicator) is more objective, quantitative, non-invasive, precise, and appropriate for varying levels of function. Studies have shown that an abnormal pattern of ABR waveforms in ASD children indicates dysfunction at the subcortical level (Russo et al., 2010a; Miron et al., 2018). More importantly, auditory brainstem function has been linked to language impairment (Banai et al., 2005; Wible et al., 2005; Johnson et al., 2007). However, those results have been contradictory, involving prolongation, shortening, and no abnormalities in the latency and/or amplitude of waveforms (Klin, 1993; Miron et al., 2017). Besides, not all ASD children have subcortical auditory deficits (Russo et al., 2010a), and the exact percentage needs to be obtained by large-sample studies. These findings suggest there are potential auditory subcortical processing-related subtypes of ASD children linked to language impairment.

Functional neuroimaging studies in the past decade have provided evidence that the cortico–subcortical network contributes to auditory, speech and language functions (Dick et al., 2014). According to the theory of corticofugal systems (Winer, 2005), the cortex may provide an inevitable contribution to the subcortex during auditory processing (Yan and Ehret, 2002; Chandrasekaran and Kraus, 2010). Furthermore, numerous studies have found abnormalities in the cortex and subcortex levels of ASD cases, and these abnormalities have been related to language impairment (Brambilla et al., 2003; Bauman and Kemper, 2005). Taken together, those findings suggest the possibility of interactions among the cortex, subcortex, and language in ASD children, but little empirical evidence is available.

In the present study, we subcategorized participants with ASD by the speech-ABR. Then, we used structural magnetic resonance imaging (sMRI) to find the neural substrates for a possible subtype of the subcortical auditory function in ASD. Finally, we explored the interactive relationship among the cortex, subcortex, and language. We hypothesized that: (i) two distinct subtypes in a population of ASD cases aged 3–6 years would have significant differences in language scores; (ii) there would be significant differences in brain structure among ASD subtypes and typically developing (TD) children; and (iii) structural differences of ASD subgroups would interact with the function of the subcortex and language in ASD children (hypothesized model see Supplementary Figure 1).

## Materials and Methods

### Ethical Approval of the Study Protocol

The study protocol was approved by the Research Ethics Board of Affiliated Shenzhen Maternity & Child Healthcare Hospital (ASMCHH; Shenzhen, China). For ASD children who met the inclusion criteria, relevant researchers informed the parents of the research content, and invited them to participate in the present study. Written informed consent was obtained from the parents of children enrolled in our study.

### Participants

Twenty-five TD and thirty ASD children between 3 and 6 years of age were recruited from department of Child Psychiatry and Rehabilitation, Affiliated Shenzhen Maternity & Child Healthcare Hospital (Guangdong, China). All ASD participants were diagnosed by child psychiatrists with extensive experience at ASMCHH, and met the criteria for ASD using the Autism Diagnostic Observational Schedule (ADOS) and Diagnostic and Statistical Manual, Fifth Edition (DSM-5) of the American Psychiatric Association (2013). Child psychiatrists recommended these children undergo magnetic resonance imaging (MRI) to exclude organic brain disorders.

All participants passed hearing screenings at birth and had good hearing sensitivity bilaterally (<25 dB HL from 500 to 4,000 Hz). Besides, all participants were screened to ensure that they met the inclusion criteria: (i) met criteria of autistic disorder using both the ADOS and DSM-5 criteria; (ii) age of 3–6 years; (iii) no diseases of the acoustic meatus or hearing disorders; (iv) no history of epilepsy or head injury; and (v) no combined other neurodevelopmental disorders. Age-matched TD participants were screened with questionnaires. Individuals with a family history of any neuropsychiatric disorder (e.g., autism, learning disability, affective disorders, schizophrenia, and epilepsy) were excluded. All parents of participants were informed of the need to add a three-dimensional (3D) scan sequence, speech-ABR, and relevant clinical evaluation.

### Clinical Evaluation

#### ADOS

The ADOS is a semi-structured, standardized interaction-and-observation tool that measures autism symptoms for individuals with possible autism or other pervasive developmental disorders (Lord, 1989). Each module contains standard activities and materials that allow examiners to assess the developmental and language levels of participants (Stacy et al., 2012).

#### Childhood Autism Rating Scale

The Childhood Autism Rating Scale (CARS) is a behavior-observation instrument which differentiates people with ASD from those with other developmental disorders (Schopler et al., 1980; Chlebowski et al., 2010). The instrument consists of 15 domains (14 domains assessing behaviors associated with autism and one domain assessing the general impressions of autism) rated on a seven-point scale from “normal” to “severely abnormal.” The total score of all domains is evaluated, which is useful as a continuous measure of autism severity.

#### Gesell Developmental Diagnosis Scale

The Gesell Developmental Diagnosis Scale (GDDS) can be used to evaluate the developmental status of infants and young children from the age of 0 to 72 months (Westman, 1976; Raheli et al., 2009). GDDS assesses different aspects of developmental (including adaptive, gross motor, fine motor, language, and personal–social) abilities (Meinzen et al., 2010). Each participant was assigned a developmental quotient (DQ). This is the ratio between the developmental age and chronological age in each of the five specific domains. DQ ≥ 76 was considered “normal”; 55–75 denoted “mild retardation”; 40–54 indicated “moderate retardation”; ≤39 reflected “severe retardation.”

#### Speech-ABR

The stimulus comprised the five first formants of the syllable /da/, including consonant /d/ and vowel /a/. The /da/ was 40 ms in duration, and synthesized by a Klatt (1980) synthesizer at a rate of 10 kHz. Laterality can affect the speech-ABR, so the stimulus was presented monaurally (right ear) (Hornickel and Skoe, 2009). The stimulus intensity was 80 dB sound pressure level with a presentation rate of 10.9 stimuli/s through headphones. The default stimulus was provided using Biological Marker of Auditory Processing (BioMARK) software (Boise, ID, United States). Recording of the speech-ABR took place in an electrophysiology room at ASMCHH. The Navigator PRO system (Bio-logic Systems, Mundelein, IL, United States) and BioMARK software were used to record the speech-ABR. Three sweeps of 3000 stimuli were recorded and grand-averaged for each participant to improve the signal-to-noise ratio. The parameters used to obtain the speech-ABR have been used in our previous study (Chen et al., 2019).

##### Criteria for subtypes

**FIGURE 1:**
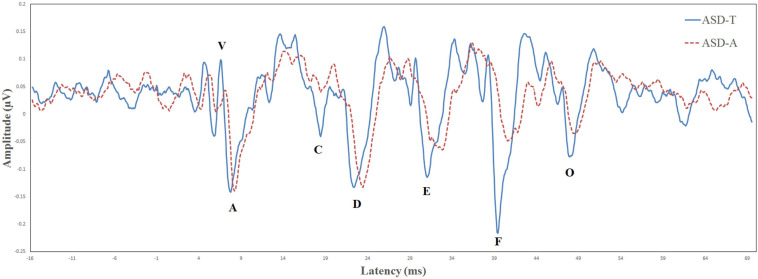
Comparison of the brainstem response to speech in the ASD-T group (solid line) and ASD-A group (dotted line).

Biological Marker of Auditory Processing is a testing protocol developed by Auditory Neuroscience Laboratory of Northwestern University (Evanston, IL, USA). The parameters evaluated for the speech-ABR were the: latency of wave-V; latency of wave-A; V/A slope; first formant frequencies; higher frequencies (Billiet and Bellis, 2011; Sanfins et al., 2015). The BioMARK score was obtained as a composite score derived from all five parameters that it assessed. There were different scoring criteria in different age groups. At the age of 3–4 years, a score of ≤3 was considered to be “normal” and a score from 4 to 22 was considered “abnormal.” At the age of 5–12 years, a score of ≤7 was considered “normal” and a score from 8 to 22 was considered “abnormal.” According to whether the speech-ABR was normal or abnormal, ASD participants were divided into the ASD-atypical (ASD-A) group or ASD-typical (ASD-T) group. According to BioMARK scores, ASD participants were divided into the ASD-A group (*n* = 15) and ASD-T group (*n* = 15). The BioMARK score was normal for all TD children. The ASD-T, ASD-A, and TD groups were matched on age. Relevant demographic information is shown in Table 1. The grand-average waveforms of the speech-ABR between the two subgroups were illustrated in Figure 1.

**TABLE 1:** Demographic information of ASD and TD participants.

	ASD	ASD-T	ASD-A	TD	*F*	*p*
Number	29	15	14	25		
Male:female	27:2	13:2	14:0	17:8		
Age (years)	4.51 ± 1.20	4.17 ± 1.00	4.71 ± 1.22	4.63 ± 0.56	1.332	0.270

*Data are the mean ± SD.*

#### Acquisition of MRI Data

For ASD participants who could cooperate to undergo MRI, 10% chloral hydrate (0.75 mL/kg, p.o.) was used for sedation. All ASD children were sedated for MRI according to the sedation protocol set by the Department of Radiology of ASMCHH. The imaging data of TD children were collected during natural sleep at night without use of sedation. High-resolution T1-weighted MRI sequences were obtained on a 1.5-T Magnetom Symphony Maestro Class Syngo MR 2002B scanner (Siemens, Munich, Germany) with echo time = 3.30 ms, repetition time = 10 ms, flip angle = 15°, 180 slices, and in-plane voxel size = 1 mm × 1 mm.

Following visual assessment of MRI data and output from the FreeSurfer image analysis suite^1^, the raw data from one ASD participant which were of insufficient quality were excluded. Hence, the final analysis comprised data from 29 ASD cases and 25 TD participants.

#### Processing of MRI Data

T1-weighted images were processed using FreeSurfer v6.0.0. Cortical reconstruction for each participant was examined slice-by-slice to identify inaccuracies in surface placement. Inaccuracies were corrected or excluded by a single experienced FreeSurfer user. The details of these procedures are described elsewhere (Wallace et al., 2013). Surface area, cortical thickness, and cortical volume were measured automatically at each FreeSurfer surface vertex. The local gyrification index is a 3D surface-based measure of the degree of cortical folding (which is the ratio of the cortical surface area within the sulcal folds relative to the amount of cortex on the outer visible cortex). The local gyrification index was measured using an added flag to the FreeSurfer reconstruction-processing stream (Schaer et al., 2008).

### Statistical Analyses

Group differences in the latency and amplitude of speech-ABR were assessed using one-way analysis of variance (ANOVA) with the diagnostic group as a three-level (ASD-A, ASD-T and TD) factor adjusted for multiple comparisons. If the main effect of the group was significant, *post hoc* pairwise comparisons were conducted using the Bonferroni correction. The complete characterization and compare of speech-ABR among three group were reported in Table 2. A between-group analysis using a two-sample *t*-test was undertaken to detect differences in scores for the ADOS, CARS, GDDS, and speech-ABR between the ASD-A group and ASD-T group. A two-step general linear model was used to estimate the difference in surface area, cortical thickness, cortical volume and the local gyrification index between two groups, including ASD and TD, ASD-A and TD, ASD-T and TD, and ASD-A and ASD-T. Surface area, cortical thickness, and cortical volume were smoothed using a 10-mm full-width at half maximum (FWHM) 2D Gaussian kernel. The local gyrification index measure is intrinsically smooth, so data were smoothed at 5-mm FWHM. To provide stringent criteria to minimize false-positive results, all analyses were set at *p* < 0.01 (corrected for multiple comparisons using Monte Carlo stimulations) (Hagler et al., 2006). Clusters identified in the Monte Carlo stimulations were used as regions of interest which the value of the brain structure was extracted from. A Fisher transformation was applied to improve the normality of the correlation coefficient (Lowe et al., 1998). Based on all ASD participants having different degrees of language impairment, Pearson correlations were undertaken in all ASD participants to investigate the relationship among brain structure, speech-ABR waveforms, and clinical assessments.

**TABLE 2:** Latency and amplitude of speech-ABR waveforms recorded in ASD subgroups and the TD group.

Wave		ASD-T	ASD-A	TD	ANOVA		*Post hoc*		
					*F*	*p*	ASD-T vs. ASD-A	ASD-T vs. TD	ASD-A vs. TD
V	Latency (ms)	6.57 ± 0.24	7.23 ± 0.46	6.70 ± 2.45	16.07	0.000*	0.000**	0.434	0.000**
	Amplitude (μν)	0.11 ± 0.07	0.04 ± 0.06	0.11 ± 0.06	6.74	0.003*	0.009**	1.000	0.005**
A	Latency (ms)	7.52 ± 0.34	8.42 ± 0.57	7.67 ± 0.27	20.18	0.000*	0.000**	0.739	0.000**
	Amplitude (μν)	−0.21 ± 0.05	−0.22 ± 0.08	−0.22 ± 0.08	0.03	0.966	1.000	1.000	1.000
C	Latency (ms)	18.27 ± 0.31	18.64 ± 1.05	18.45 ± 0.50	1.11	0.338	0.433	1.000	1.000
	Amplitude (μν)	−0.09 ± 0.05	−0.03 ± 0.05	−0.13 ± 0.21	1.97	0.151	1.000	1.000	0.163
D	Latency (ms)	22.27 ± 0.41	23.17 ± 0.52	22.49 ± 0.63	8.41	0.001*	0.001**	0.467	0.010**
	Amplitude (μν)	−0.18 ± 0.07	−0.21 ± 0.06	−0.18 ± 0.08	1.80	0.176	1.000	0.855	0.214
E	Latency (ms)	31.10 ± 0.55	31.36 ± 0.80	31.16 ± 0.77	0.51	0.602	1.000	1.000	1.000
	Amplitude (μν)	−0.20 ± 0.09	−0.14 ± 0.05	−0.22 ± 0.11	2.39	0.102	0.370	1.000	0.109
F	Latency (ms)	39.57 ± 0.59	40.56 ± 0.88	39.54 ± 0.59	11.00	0.000*	0.001**	1.000	0.000**
	Amplitude (μν)	−0.27 ± 0.09	−0.15 ± 0.05	−0.22 ± 0.14	4.50	0.016	0.013	0.522	0.149
O	Latency (ms)	47.67 ± 0.86	48.86 ± 1.27	48.06 ± 0.39	7.52	0.001*	0.001**	0.452	0.019**
	Amplitude (μν)	−0.14 ± 0.06	−0.13 ± 0.04	−0.18 ± 0.17	1.11	0.338	1.000	0.787	0.598

*Data are the mean ± SD.*

SD, standard deviation.

*Significant using the threshold 0.05/14 = 0.004, adjusted for multiple comparisons.

**Significant using the threshold 0.05, adjusted for the Bonferroni correction.

#### Mediation Effect

According to the result of correlation analyses, we further explored whether the auditory brainstem function moderated the relationship between the brain structure and language ability of all ASD participants controlled for age and sex. The PROCESS macro program within SPSS (IBM, Armonk, NY, United States) designed by Hayes (2013) was used to measure the mediating or moderating effect. Within PROCESS, “model 4” was selected and the confidence interval was set to 95%. In the moderation model, the surface area of the left rostral middle frontal gyrus (lRMFG) was entered as the predictor (*X*), language score of the GDDS as the outcome (*Y*), and the amplitude of wave-V as the moderator (*M*). Statistical tests were evaluated at *p* < 0.05 (two-tailed).

## Results

### Clinical Evaluation of ASD Subgroups

As shown in Table 3, ASD subgroups did not differ in scores for the ADOS or CARS (Table 3). Two subgroups of ASD participants were mildly retarded and did not differ in gross-motor, fine-motor, or adaptive functions. In GDDS-language, the score of the ASD-A group was lower than that of the ASD-N group (*t* = 2.425, *p* = 0.025).

**TABLE 3:** Clinical characteristics of ASD subgroups.

	ASD-T	ASD-A	*t*	*p*
CARS-score	31.58 ± 1.52	31.50 ± 1.30	0.114	0.910
ADOS-communication	5.50 ± 0.97	5.11 ± 2.26	0.497	0.626
ADOS-social interaction	7.80 ± 1.69	6.78 ± 1.48	1.396	0.181
ADOS-score	13.30 ± 2.45	11.89 ± 3.41	1.044	0.311
GDDS-adaptive	65.07 ± 17.59	59.55 ± 14.64	0.847	0.406
GDDS-gross motor	72.07 ± 9.55	69.22 ± 10.26	0.679	0.505
GDDS-fine motor	64.07 ± 13.18	61.22 ± 5.97	0.606	0.551
GDDS-language	59.43 ± 17.67	45.71 ± 10.37	2.425	0.025*
GDDS-social	61.50 ± 13.18	56.22 ± 9.62	1.034	0.313

*Data are the mean ± SD.*

CARS, Child Autism Rating Scale; ADOS, Autism Diagnostic Observation Scale; GDDS, Gesell Developmental Diagnosis Scale; SD, standard deviation. *p < 0.05.

### Differences in Brain Structure Among the Groups

**FIGURE 2:**
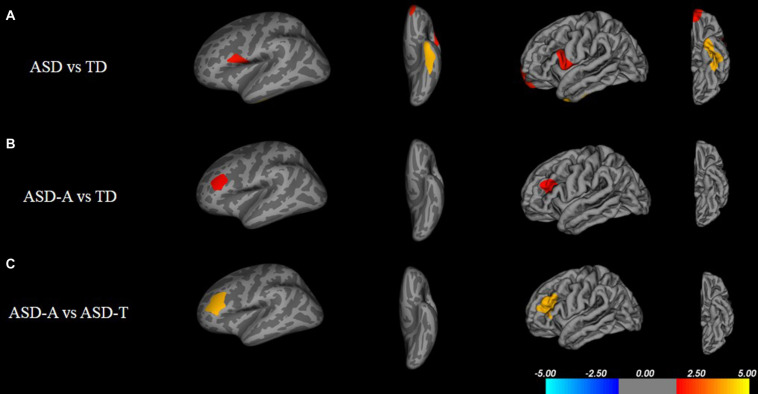
Inflated and pial surface maps (dark gray, sulci; light gray, gyri) of the left hemispheres showing increasing surface area in **(A)** fusiform, frontal pole and pars opercularis in the ASD group compared with that in the TD group, **(B,C)** rostral middle frontal gyrus in the ASD-A group compared with that in the TD group and ASD-T group. Significance threshold was set at *p* < 0.01 (cluster-corrected).

**FIGURE 3:**
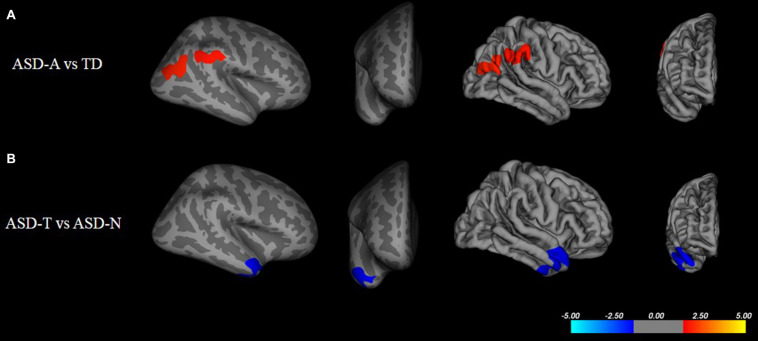
Inflated and pial surface maps (dark gray, sulci; light gray, gyri) of the right hemispheres showing a decreasing local gyrification index in **(A)** inferior parietal and supramarginal areas in the TD group compared with that in the ASD-A group, **(B)** superior temporal gyrus (STG) in the ASD-A group compared with that in the ASD-T group. Significance threshold was set at *p* < 0.01 (cluster-corrected).

There were significant (cluster-corrected *p* < 0.01) differences between the TD group and ASD group for cortical thickness, cortical volume and surface area (Supplementary Table 1 and Figure 2). Compared with the TD group, the ASD-T group and ASD-A group showed a significant difference in surface area, cortical volume, cortical thickness, and the local gyrification index (Table 4). Surface area of lRMFG in ASD-A group was larger than that in the ASD-T group and TD group (Figure 2 and Table 4). The local gyrification index of the right superior temporal gyrus (rSTG) in the ASD-A group was decreased significantly compared with that in the ASD-T group (Figure 3 and Table 4).

**TABLE 4:** Clusters of significant differences in cortical morphometry.

Group	Measure	Size (mm^2^)	*X*	*Y*	*Z*	Number of vertices	Peak region
ASD-A vs. ASD-T	Area	1067.46	–38.4	36.1	28.7	1747	L rostral middle frontal
	Local gyrification index	859.3	47.5	5	–27.2	1271	R superior temporal
ASD-T vs. TD	Thickness	5114.47	–26.3	23.8	–6	9550	L lateral orbitofrontal
		769.09	–19.9	–88	–7.4	789	L lateral occipital
		1122.41	44	–12.8	20.7	3031	R postcentral
		1098.71	27.7	57.8	–9.5	1551	R rostral middle frontal
		1056.15	8.3	37	–3.9	1711	R rostral anterior cingulate
		1015.37	31	–41.1	–9	1822	R parahippocampal
		602.63	37	–84	15.9	830	R inferior parietal
		550.12	44	–67.7	7	1079	R inferior parietal
		548.19	45.1	–37.2	17.7	1110	R supramarginal
	Area	728.78	–55.3	–12.4	31.3	1665	L postcentral
	Volume	458.44	–39.8	–68.1	0	783	L lateral occipital
		407.34	–55.3	–12.4	31.3	888	L postcentral
		1231	1231	–67.7	7	1901	R inferior parietal
		540.54	29.7	–45.7	–15.4	943	R fusiform
	Local gyrification index	1593.53	–12	–67.1	34.7	2723	L precuneus
ASD-A vs. TD	Thickness	1595.18	–26.3	23.8	–6	4144	L lateral orbitofrontal
		1022.82	–12.8	–11.1	67.7	2159	L superior frontal
		693.74	–7.3	37.5	13.5	1199	L rostral anterior cingulate
		528.3	–38.1	50	–3.4	732	L rostral middle frontal
		509.2	–4.1	–33.4	30.5	1282	L isthmus cingulate
		456.39	–28.8	–47.9	–5.4	925	L lingual
		1378.09	14.2	–65.9	–3	2041	R lingual
		1151.45	37.1	–13	1.8	3134	R insula
		834.91	3.3	–32.8	65.1	1974	R paracentral
		733.2	12.8	–52.3	41.4	1846	R precuneus
		564.84	8.3	37	–3.9	1067	R rostral anterior cingulate
		558.42	32.5	35.1	–6.7	844	R lateral orbitofrontal
	Area	760.35	–37.3	23.6	24.8	1337	L rostral middle frontal
	Volume	1289.21	–37.3	23.6	24.8	2007	L rostral middle frontal
		940.3	–13.4	–91.2	3.8	1497	L pericalcarine
		675.25	–58.1	–20.9	–14.8	1398	L middle temporal
		847.58	44	–67.7	7	1395	R inferior parietal
	Local gyrification index	1091.79	45	–60.1	21.3	1869	R inferior parietal
		1030.32	55.1	–38.4	27.5	2179	R supramarginal

### Correlations Among Brain Structure, Speech-ABR and Clinical Scores in ASD Group

We also analyzed the potential link among the speech-ABR, brain-structural and clinical-assessment data. Surface area of lRMFG was related to the latency of waves V and A, and amplitude of wave-V (Table 5). Local gyrification index of rSTG was related to the latency of waves V and A. GDDS-language scores were related to the amplitude of wave-V and latency of wave-A.

**TABLE 5:** Association between speech-ABR waveforms and brain structure and GDDS-language characteristics in ASD participants.

	Surface area-lRMFG		Local gyrification index-rSTG		GDDS-language	
	*r*	*p*	*r*	*p*	*r*	*p*
Wave-V latency	0.452	0.014*	–0.428	0.023*	–0.351	0.067
Wave-V amplitude	–0.533	0.003*	0.019	0.922	0.551	0.002*
Wave-A latency	0.402	0.031*	–0.533	0.003*	–0.479	0.010*
Wave-A amplitude	–0.067	0.729	0.003	0.989	0.066	0.738
GDDS-language	–0.262	0.178	–0.102	0.612	–	–

lRMFG, left rostral middle frontal gyrus; rSTG, right superior temporal gyrus; GDDS, Gesell Developmental Diagnosis Scale.

*p < 0.05.

### Mediation Effect Among Regional Surface Area, Language Scores and Amplitude of Wave

**FIGURE 4:**
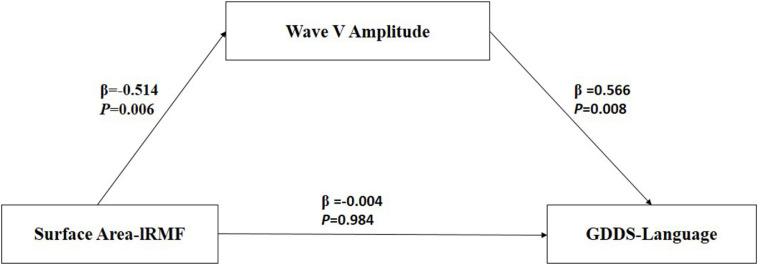
Mediation model among surface area-lRMFG (predictor), Wave-V amplitude (mediation), and GDDS-language (outcome). β, standardized regression coefficient. Surface area-lRMFG, surface area of left rostral middle frontal gyrus; GDDS-language, language scores of the Gesell Developmental Diagnosis Scale.

Table 6 presents the results of the mediation model. Figure 4 shows the regression coefficient for each pathway from surface area of lRMFG to language-GDDS in the mediation model. There was a significant negative association between surface area of lRMFG and the wave-V amplitude (β = −0.514, *p* = 0.006). There was a significant positive association between the wave-V amplitude and GDDS-language (β = 0.566, *p* = 0.008). The bootstrap procedure revealed significant indirect effects between surface area of lRMFG and GDDS-language controlled for age and sex [β = −0.013, SE = 0.007, BC 95%CI (−0.028, −0.002)].

**TABLE 6:** Direct and indirect effects between surface area of lRMFG and GDDS-language mediated by the amplitude of wave-V.

	Product of coefficients		BC 95% bootstrap CI	
	β	SE	Boot LL CI	Boot UL CI
Direct effect	−0.004	0.009	−0.0184	0.0180
Indirect effect	−0.013	0.007	−0.0280	−0.0016

*Mediation model controlled for age and sex.*

*“BC 95% CI” refers to the bias-corrected confidence intervals. The mediate effect is significant at the 0.05 level of significance.*

*β, standardized regression coefficient; SE, standard error; CI, confidence interval; lRMFG, left rostral middle frontal gyrus; GDDS-language, language scores for the Gesell Developmental Diagnosis Scale.*

## Discussion

This is the first study to classify ASD children into two subtypes based on function of auditory brainstem and to discover neural substrates for the possible subtypes.

Our study elicited four main findings. First, the language score of the ASD-A group was lower than that of the ASD-T group. Second, compared with the TD group, the ASD subgroups showed significant differences in brain structure. Surface area of lRMFG in the ASD-A group was larger, and the local gyrification index of rSTG in the ASD-A group was decreased significantly, compared with those in the ASD-T group. Third, GDDS-language and surface area of lRMFG were correlated to the wave-A amplitude in ASD. Finally, analysis revealed surface area of lRMFG to have an indirect effect on language *via* the wave-V amplitude in ASD children.

Autism spectrum disorder subgroups defined in the present study showed a significant difference in the speech-ABR at preschool age. The ASD-T group exhibited age-appropriate subcortical auditory processing function. In contrast, the ASD-A group showed the latency of speech-ABR waves to be longer and/or the amplitude to be smaller compared with those in the TD group and ASD-T group. Besides, ASD subtypes showed a significant difference in language but not in other aspects or symptom severity. Consistent with our previous research showing that the speech-ABR is related to language ability (Chen et al., 2019), children with an efficient speech-ABR had better language ability. Our study indicated that the speech-ABR could be a clinical-assessment tool to predict the language ability of ASD children at preschool age. Combined with findings that early language ability is an important predictive factor for later outcomes of ASD (Szatmari et al., 2010; Tager-Flusberg and Kasari, 2013), ASD children with a normal speech-ABR may have a better outcome compared with abnormal ones. Taken together, these data suggest that auditory brainstem function not only has a crucial role in language, but also in the long-term outcome of ASD children.

We found differences in brain structure not only between TD children and ASD children, but also among ASD subtypes. Consistent with other studies, we found an aberrant cortical structure in ASD children, including cortical thickness, surface area, and cortical volume (Blackmon et al., 2016; Patriquin et al., 2016). Besides, differences in the local gyrification index were observed only between ASD subtypes and TD, and not ASD and TD. Combined with contradictory results in the neuroimaging of ASD (Pua et al., 2017), ASD may be composed of different subtypes which exist differences in brain anatomy. Hence, comparison of ASD and TD before correct differentiation of subtypes will lead to unstable and unrepeatable research results.

Moreover, we found a significant difference in brain structure between ASD subtypes, which may provide neuroimaging evidence for subtype classification. We found a larger regional surface area (lRMFG) in the ASD-A group compared with that in the ASD-T group and TD group. This result indicated that an increase in surface area of lRMFG was a characteristic structural change in the ASD-A subtype. The RMFG, as part of the dorsolateral prefrontal cortex (DLPFC), is related to the function of language, as discovered in various lesion studies (Chapman et al., 1992, 1998). Patients with left-DLPFC lesions have been found to use fewer complex sentences and to perseverate on the first proposition (Carl et al., 2012). These symptoms can also occur in some ASD children who use simple sentences and “chatter” on a topic. Besides, a lower local gyrification index of the right STG was found in the ASD-A group. The traditional view is that the STG is involved primarily in auditory processing, including language (Gernsbacher and Kaschak, 2003; Martin, 2003). However, recent studies have reported that the STG is not only involved in auditory processing, but is also implicated in social cognition (Bigler et al., 2007; Ethofer et al., 2013). Taken together, the differences in brain structure between ASD subtypes are involved in the cortex related to auditory and language, but also in the high-order cortex related to society. The differences in altered subtypes in the brain may be one of the important reasons for ASD heterogeneity.

Further analyses revealed that surface area of lRMFG had an indirect effect on language by altering the wave-V amplitude in ASD children. Combined with the previous findings showing that the neural generators of waves V and A may be the inferior colliculus (Song et al., 2008), one parsimonious explanation is that the lRMFG targeting the inferior colliculus mediates the language of ASD children through corticofugal neurons. As a crucial mechanism of neuromodulation, physiological studies have demonstrated that these descending pathways can affect several aspects of subcortical function (Villa et al., 1991; Diamond et al., 1992; Ma and Suga, 2001). Studies have demonstrated language impairment to be related to cortical and/or subcortical dysfunctions in ASD children (Groen et al., 2008; Russo et al., 2010a). Our findings suggest that cortical deficits may impair language ability in children with ASD by causing subcortical dysfunction at preschool age.

Using subtypes may be the most useful method for specific intervention in ASD. Experience-dependent mechanisms drive the plasticity of the auditory brainstem, which can be improved by auditory training (Hayes et al., 2003; Russo et al., 2005) and language experience (Krishnan et al., 2004, 2005). Skoe et al. (2015) proposed the corticofugal system nears maturation stabilization around the age of 8–11 years. Therefore, we postulate that ASD children with an abnormal speech-ABR should undergo related auditory and language training as soon as possible, especially at preschool age. The Fast ForWord language-training program has been found to improve auditory-brainstem and cortical responses in ASD children (Russo et al., 2010b). Few intervention studies on ASD have been done. However, based on behavioral and genetic similarities (Herbert and Kenet, 2007; Smith, 2007) in language impairment between ASD and language-based learning disorders (Rapin and Dunn, 2003; Cardy et al., 2005), intervention programs of language-based learning disorders to improve auditory-brainstem function could be conducted in ASD children with an abnormal speech-ABR. In addition, the subtype-based method may be useful as a prognostic biomarker in ASD children. A stable ABR is associated with heightened language abilities (Hornickel and Kraus, 2013). Previously, we found the auditory brainstem to be impaired and immature in preschool children with ASD (Chen et al., 2019). We speculated the ASD children with an abnormal speech-ABR indicates impairment of the related brain region and poor language ability, which may lead to a poor prognosis. In the future, we will examine if an abnormal speech-ABR is associated with a worse long-term outcome compared with normal ones. Overall, ASD children should undergo a speech-ABR after 3 years of age for use as a subtype and prognostic biomarker, and a subtype-specific intervention should be employed for a better outcome.

In interpreting our results, some limitations should be considered. First, our study had a relatively small sample size. There were only two female participants in this study, and both were in the ASD-A. This study does not exclude the potential of gender factors for subtypes. Our future studies will recruit more children with ASD who meet the criteria for an appropriate gender ratio. Second, MRI was done on a 1.5-T system, but several recent studies have used 3-T MRI scanners. MRI scanners working at 3 T have a higher field strength and increased signal-to-noise ratio compared with those using a 1.5-T MRI scanner. In future work, we will use a 3.0-T MRI scanner to acquire higher spatial resolution data. Finally, intelligence and language ability are highly correlated. Although there were no significant differences in overall developmental levels between the two subtypes. It is possible that there were significant differences in intelligence levels. Our future studies will investigate whether intellectual factors affect subcortical auditory processing in ASD.

## Conclusion

This is first study to distinguish ASD children by auditory brainstem function and to explore neural evidence for identification of potential subtypes. There were significant differences between the ASD group and TD group in surface area, cortical volume, and cortical thickness. Compared with the ASD-T group, the ASD-A group showed a lower score in GDDS-language, increased surface area of lRMFG, and decreased local gyrification index of rSTG. GDDS-language and surface area of lRMFG were correlated with the wave-A amplitude. Surface area of lRMFG had an indirect effect on the performance of language by altering the wave-V amplitude. Thus, cortical deficits may impair language ability in children with ASD by causing subcortical dysfunction at preschool age. These evidences support dysfunction of the auditory brainstem as a potential subtype of ASD. Besides, this subtype-based method may be useful for various clinical applications (e.g., prognosis and subtype-specific intervention).

## Data Availability Statement

The original contributions presented in the study are included in the article/Supplementary Material, further inquiries can be directed to the corresponding author/s.

## Ethics Statement

The studies involving human participants were reviewed and approved by the Research Ethics Board of Affiliated Shenzhen Maternity & Child Healthcare Hospital. Written informed consent to participate in this study was provided by the participants’ legal guardian/next of kin.

## Author Contributions

JC, ZP, and GW conceived the study, prepared the data, analyzed the data, and drafted and revised the manuscript. BL, JG, and MH helped to analyze the data and helped to draft the manuscript. XK and CL participated in the study design and helped to draft and revise the manuscript. All authors approved the final version of the manuscript.

## Conflict of Interest

The authors declare that the research was conducted in the absence of any commercial or financial relationships that could be construed as a potential conflict of interest.

## Footnotes

1 http://surfer.nmr.mgh.harvard.edu/

## Supplementary Material

Supplementary Figure1 Hypothesized model among cortex, subcortex, and language in ASD preschool children.

Supplementary Table 1 Cluster of significant differences in cortical morphometry between the TD group and ASD group.

## Abbreviations

ASD: autism spectrum disorder
ABR: auditory brainstem response
speech-ABR: speech-evoked auditory brainstem response
MRI: magnetic resonance imaging
ADOS: Autism Diagnostic Observational Schedule
CARS: Childhood Autism Rating Scale
GDDS: Gesell Developmental Diagnosis Scale
DQ: developmental quotient
BioMARK: Biological Marker of Auditory Processing
lRMFG: left rostral middle frontal gyrus
rSTG: right superior temporal gyrus.

## References

[B1] American Psychiatric Association (2013). *Diagnostic and Statistical Manual of Mental Disorders*, 5th Edn. Washington, DC: American Pshychartic Association.

[B2] Anderson D. K. Catherine L. Susan R. Dilavore P. S. Cory S. Audrey T. (2007). Patterns of growth in verbal abilities among children with autism spectrum disorder. *J. Consult. Clin. Psychol.* 75:594. 10.1037/0022-006X.75.4.594 17663613

[B3] Anderson S. Skoe E. Chandrasekaran B. Kraus N. (2010). Neural timing is linked to speech perception in noise. *J. Neurosci.* 30 4922–4926. 10.1523/jneurosci.0107-10.2010 20371812 PMC2862599

[B4] Banai K. Nicol T. Zecker S. G. Kraus N. (2005). Brainstem timing: implications for cortical processing and literacy. *J. Neurosci.* 25 9850–9857. 10.1523/JNEUROSCI.2373-05.2005 16251432 PMC6725554

[B5] Bauman M. L. Kemper T. L. (2005). Neuroanatomic observations of the brain in autism: a review and future directions. *Int. J. Dev. Neurosci.* 23 183–187. 10.1016/j.ijdevneu.2004.09.006 15749244

[B6] Bigler E. D. Mortensen S. Neeley E. S. Ozonoff S. Krasny L. Johnson M. (2007). Superior temporal gyrus, language function, and autism. *Dev. Neuropsychol.* 31 217–238. 10.1080/87565640701190841 17488217

[B7] Billiet C. R. Bellis T. J. (2011). The relationship between brainstem temporal processing and performance on tests of central auditory function in children with reading disorders. *J. Speech Lang. Hear. Res.* 54 228–242. 10.1044/1092-4388(2010/09-0239) 20689038

[B8] Bitsika V. Sharpley C. F. Orapeleng S. (2008). An exploratory analysis of the use of cognitive, adaptive and behavioural indices for cluster analysis of ASD subgroups. *J. Intell. Disabil. Res.* 52 973–985. 10.1111/j.1365-2788.2008.01123.x 19017167

[B9] Blackmon K. Ben-Avi E. Wang X. Pardoe H. R. Di Martino A. Halgren E. (2016). Periventricular white matter abnormalities and restricted repetitive behavior in autism spectrum disorder. *Neuroimage Clin.* 10 36–45. 10.1016/j.nicl.2015.10.017 26693400 PMC4660377

[B10] Brambilla P. Hardan A. di Nemi S. U. Perez J. Soares J. C. Barale F. (2003). Brain anatomy and development in autism: review of structural MRI studies. *Brain Res. Bull.* 61 557–569. 10.1016/j.brainresbull.2003.06.001 14519452

[B11] Cardy J. E. O. Flagg E. J. Roberts W. Brian J. Roberts T. P. J. N. (2005). Magnetoencephalography identifies rapid temporal processing deficit in autism and language impairment. *Neuroreport* 16 329–332. 10.1097/00001756-200503150-00005 15729132

[B12] Carl C. Karen L. Jennifer M. Frank K. Jordan G. J. N. (2012). Discourse production following injury to the dorsolateral prefrontal cortex. *Neuropsychologia* 50 3564–3572. 10.1016/j.neuropsychologia.2012.09.005 22982512

[B13] Chandrasekaran B. Kraus N. (2010). The scalp-recorded brainstem response to speech: neural origins and plasticity. *Psychophysiology* 47 236–246. 10.1111/j.1469-8986.2009.00928.x 19824950 PMC3088516

[B14] Chapman S. B. Culhane K. A. Levin H. S. Harward H. Mendelsohn D. Ewing-Cobbs L. (1992). Narrative discourse after closed head injury in children and adolescents. *Brain Lang.* 43 42–65. 10.1016/0093-934x(92)90020-f 1643511

[B15] Chapman S. B. Levin H. S. Wanek A. Weyrauch J. Kufera J. (1998). Discourse after closed head injury in young children. *Brain Lang.* 61 420–449. 10.1006/brln.1997.1885 9570872

[B16] Chen J. Liang C. Wei Z. Cui Z. Kong X. Dong C. J. (2019). Atypical longitudinal development of speech-evoked auditory brainstem response in preschool children with autism spectrum disorders. *Autism Res.* 12 1022–1031. 10.1002/aur.2110 31025832

[B17] Chlebowski C. Green J. A. Barton M. L. Fein D. (2010). Using the childhood autism rating scale to diagnose autism spectrum disorders. *J. Autism Dev. Disord.* 40 787–799. 20054630 10.1007/s10803-009-0926-x PMC3612531

[B18] De Fossé L. Hodge S. M. Makris N. Kennedy D. N. Caviness V. S. Mcgrath L. (2010). Language-association cortex asymmetry in autism and specific language impairment. *Ann. Neurol.* 56 757–766. 10.1002/ana.20275 15478219

[B19] Deboth K. K. Reynolds S. (2017). A systematic review of sensory-based autism subtypes. *Res. Autism Spectrum Disord.* 36 44–56. 10.1016/j.rasd.2017.01.005

[B20] Diamond M. E. Armstrong-James M. Budway M. J. Ebner F. F. (1992). Somatic sensory responses in the rostral sector of the posterior group (POm) and in the ventral posterior medial nucleus (VPM) of the rat thalamus: dependence on the barrel field cortex. *J. Comp. Neurol.* 319 66–84. 10.1002/cne.903190108 1592906

[B21] Dick A. S. Bernal B. Tremblay P. (2014). The language connectome: new pathways, new concepts. *Neurosci.* 20 453–467. 10.1177/1073858413513502 24342910

[B22] Ethofer T. Bretscher J. Wiethoff S. Bisch J. Schlipf S. Wildgruber D. (2013). Functional responses and structural connections of cortical areas for processing faces and voices in the superior temporal sulcus. *Neuroimage* 76 45–56. 10.1016/j.neuroimage.2013.02.064 23507387

[B23] Gernsbacher M. A. Kaschak M. P. (2003). Neuroimaging studies of language production and comprehension. *Annu. Rev. Psychol.* 54 91–114. 10.1146/annurev.psych.54.101601.145128 12359916 PMC4153591

[B24] Groen W. B. Zwiers M. P. van der Gaag R. J. Buitelaar J. K. (2008). The phenotype and neural correlates of language in autism: an integrative review. *Neurosci. Biobehav. Rev.* 32 1416–1425. 10.1016/j.neubiorev.2008.05.008 18562003

[B25] Grzadzinski R. Huerta M. Lord C. (2013). DSM-5 and autism spectrum disorders (ASDs): an opportunity for identifying ASD subtypes. *Mol. Autism* 4 1–6. 10.1186/2040-2392-4-12 23675638 PMC3671160

[B26] Hagler D. J. Jr. Saygin A. P. Sereno M. I. (2006). Smoothing and cluster thresholding for cortical surface-based group analysis of fMRI data. *Neuroimage* 33 1093–1103. 10.1016/j.neuroimage.2006.07.036 17011792 PMC1785301

[B27] Hayes A. F. (2013). *Introduction to Mediation, Moderation, and Conditional Process Analysis.* New York, NY: Guilford Press, 335–337.

[B28] Hayes E. A. Warrier C. M. Nicol T. G. Zecker S. G. Kraus N. (2003). Neural plasticity following auditory training in children with learning problems. *Clin. Neurophysiol.* 114 673–684. 10.1016/s1388-2457(02)00414-5 12686276

[B29] Heaton P. Williams K. Cummins O. Happe F. (2008). Autism and pitch processing splinter skills: a group and subgroup analysis. *Autism* 12 203–219. 10.1177/1362361307085270 18308768

[B30] Herbert M. R. Kenet T. (2007). Brain abnormalities in language disorders and in autism. *Pediatr. Clin. North Am.* 54 563–583. 10.1016/j.pcl.2007.02.007 17543910

[B31] Hornickel J. Kraus N. (2013). Unstable representation of sound: a biological marker of dyslexia. *J. Neurosci.* 33 3500–3504. 10.1523/JNEUROSCI.4205-12.2013 23426677 PMC3599785

[B32] Hornickel J. Skoe E. N. (2009). Subcortical laterality of speech encoding. *Audiol. Neurotol.* 14 198–207. 10.1159/000188533 19122453 PMC2806639

[B33] Johnson K. L. Nicol T. G. Zecker S. G. Kraus N. (2007). Auditory brainstem correlates of perceptual timing deficits. *J. Cogn. Neurosci.* 19 376–385. 10.1162/jocn.2007.19.3.376 17335387

[B34] Kjelgaard M. M. Tager-Flusberg H. (2001). An investigation of language impairment in autism: implications for genetic subgroups. *Lang. Cogn. Process.* 16 287–308. 10.1080/01690960042000058 16703115 PMC1460015

[B35] Klatt D. (1980). Software for cascade/parallel formant synthesizer. *J. Acoustical Soc. Am.*, 67 971–975. 10.1121/1.383940

[B36] Klin A. (1993). Auditory brainstem responses in autism: brainstem dysfunction or peripheral hearing loss? *J. Autism Dev. Disord.* 23 15–35. 10.1007/BF01066416 8463195

[B37] Krishnan A. Xu Y. Gandour J. Cariani P. (2005). Encoding of pitch in the human brainstem is sensitive to language experience. *Brain Res. Cogn. Brain Res.* 25 161–168. 10.1016/j.cogbrainres.2005.05.004 15935624

[B38] Krishnan A. Xu Y. Gandour J. T. Cariani P. A. (2004). Human frequency-following response: representation of pitch contours in Chinese tones. *Hear. Res.* 189 1–12. 10.1016/S0378-5955(03)00402-7 14987747

[B39] Lombardo M. V. Pierce K. Eyler L. T. Barnes C. C. Ahrens-Barbeau C. Solso S. (2015). Different functional neural substrates for good and poor language outcome in autism. *Neuron* 86 567–577. 10.1016/j.neuron.2015.03.023 25864635 PMC4610713

[B40] Lord C. O. (1989). Austism diagnostic observation schedule: a standardized observation of communicative and social behavior. *J. Autism Dev. Disord.* 19:185. 10.1007/BF02211841 2745388

[B41] Lowe M. J. Mock B. J. Sorenson J. A. (1998). Functional connectivity in single and multislice echoplanar imaging using resting-state fluctuations. *Neuroimage* 7 119–132. 10.1006/nimg.1997.0315 9558644

[B42] Ma X. Suga N. (2001). Plasticity of bat’s central auditory system evoked by focal electric stimulation of auditory and/or somatosensory cortices. *J. Neurophysiol.* 85 1078–1087. 10.1152/jn.2001.85.3.1078 11247978

[B43] Martin R. C. (2003). Language processing: functional organization and neuroanatomical basis. *Annu. Rev. Psychol.* 54 55–89. 10.1146/annurev.psych.54.101601.145201 12359917

[B44] Meinzen D. J. Wiley S. S. Choo D. I. (2010). Language performance in children with cochlear implants and additional disabilities. *Laryngoscope* 120 405–413. 10.1002/lary.20728 19950380

[B45] Miron O. Beam A. L. Kohane I. S. (2018). Auditory brainstem response in infants and children with autism spectrum disorder: a meta-analysis of wave V. *Autism Res.* 11 355–363. 29087045 10.1002/aur.1886 PMC5836986

[B46] Miron O. Beam A. L. Kohane I. S. Miron O. (2017). Auditory brainstem response in infants and children with autism: a meta-analysis. *Autism Res.* 11 355–363. 10.1002/aur.1886 29087045 PMC5836986

[B47] Patriquin M. A. Deramus T. Libero L. E. Laird A. Kana R. K. (2016). Neuroanatomical and neurofunctional markers of social cognition in autism spectrum disorder. *Hum. Brain Mapp.* 37 3957–3978. 10.1002/hbm.23288 27329401 PMC5053857

[B48] Pickles A. Anderson D. K. Lord C. (2014). Heterogeneity and plasticity in the development of language: a 17-year follow-up of children referred early for possible autism. *J. Child Psychol. Psychiatry* 55 1354–1362. 10.1111/jcpp.12269 24889883

[B49] Pua E. P. K. Bowden S. C. Seal M. L. (2017). Autism spectrum disorders: neuroimaging findings from systematic reviews. *Res. Autism Spectrum Disord.* 34 28–33. 10.1016/j.rasd.2016.11.005

[B50] Raheli D. Gustavo M. Liat B. S. Dorit L. Pick C. G. Tally L. S. (2009). Developmental outcome of children with enlargement of the cisterna magna identified in utero. *J. Child Neurol.* 24 1486–1492. 10.1177/0883073808331358 19240044

[B51] Rapin I. Dunn M. (2003). Update on the language disorders of individuals on the autistic spectrum. *Brain Dev.* 25 166–172. 10.1016/s0387-7604(02)00191-2 12689694

[B52] Russo N. Nicol T. Trommer B. Zecker S. Kraus N. (2010a). Brainstem transcription of speech is disrupted in children with autism spectrum disorders. *Dev. Sci.* 12 557–567. 10.1111/j.1467-7687.2008.00790.x 19635083 PMC2718770

[B53] Russo N. M. Hornickel J. Nicol T. Zecker S. Kraus N. (2010b). Biological changes in auditory function following training in children with autism spectrum disorders. *Behav. Brain Funct.* 6:60. 10.1186/1744-9081-6-60 20950487 PMC2965126

[B54] Russo N. M. Nicol T. G. Zecker S. G. Hayes E. A. Kraus N. (2005). Auditory training improves neural timing in the human brainstem. *J. Behav. Brain Res.* 156 95–103. 10.1016/j.bbr.2004.05.012 15474654

[B55] Russo N. M. Skoe E. Trommer B. Nicol T. Zecker S. Bradlow A. (2008). Deficient brainstem encoding of pitch in children with autism spectrum disorders. *Clin. Neurophysiol. Offic. J. Int. Feder. Clin. Neurophysiol.* 119 1720–1731. 10.1016/j.clinph.2008.01.108 18558508 PMC2536645

[B56] Sacco R. Lenti C. Saccani M. Curatolo P. Manzi B. Bravaccio C. (2012). Cluster analysis of autistic patients based on principal pathogenetic components. *Autism Res.* 5 137–147. 10.1002/aur.1226 22431251

[B57] Sanfins M. D. Borges L. R. Ubiali T. Colella-Santos M. F. (2015). Speech auditory brainstem response (speech ABR) in the differential diagnosis of scholastic difficulties. *Braz. J. Otorhinolaryngol.* 83 31–40. 10.1016/j.bjorl.2015.05.014 PMC9444797 26631329

[B58] Schaer M. Cuadra M. B. Tamarit L. Lazeyras F. Eliez S. Thiran J. P. (2008). A surface-based approach to quantify local cortical gyrification. *IEEE Trans. Med. Imaging* 27 161–170. 10.1109/TMI.2007.903576 18334438

[B59] Schopler E. Reichler R. J. Devellis R. F. Daly K. (1980). Toward objective classification of childhood autism: Childhood Autism Rating Scale (CARS). *J. Autism Dev. Disord.* 10 91–103. 10.1007/BF02408436 6927682

[B60] Skoe E. Krizman J. Anderson S. Kraus N. (2015). Stability and plasticity of auditory brainstem function across the lifespan. *Cereb. Cortex* 25:1415. 10.1093/cercor/bht311 24366906 PMC4428291

[B61] Smith S. D. (2007). Genes, language development, and language disorders. *Ment. Retard Dev. Disabil. Res. Rev.* 13 96–105. 10.1002/mrdd.20135 17326114

[B62] Song J. H. Banai K. Kraus N. (2008). Brainstem timing deficits in children with learning impairment may result from corticofugal origins. *Audiol. Neurotol.* 13 335–344. 10.1159/000132689 18493120

[B63] Stacy S. Cristan F. Audrey T. Lisa J. David B. Christine G. (2012). The ADOS calibrated severity score: relationship to phenotypic variables and stability over time. *Autism Res.* 5 267–276. 10.1002/aur.1238 22628087 PMC3422401

[B64] Szatmari P. Bryson S. E. Boyle M. H. Streiner D. L. Duku E. (2010). Predictors of outcome among high functioning children with autism and Asperger syndrome. *J. Child Psychol. Psychiatry* 44 520–528. 10.1111/1469-7610.00141 12751844

[B65] Tager-Flusberg H. Kasari C. (2013). Minimally verbal school-aged children with autism spectrum disorder: the neglected end of the spectrum. *Autism Res.* 6 468–478. 10.1002/aur.1329 24124067 PMC3869868

[B66] Villa A. E. Rouiller E. M. Simm G. M. Zurita P. de Ribaupierre Y. de Ribaupierre F. (1991). Corticofugal modulation of the information processing in the auditory thalamus of the cat. *Exp. Brain Res.* 86 506–517. 10.1007/bf00230524 1761088

[B67] Wallace G. L. Briana R. Nathan D. Lauren K. Giedd J. N. Alex M. (2013). Increased gyrification, but comparable surface area in adolescents with autism spectrum disorders. *Brain J. Neurol.* 136 1956–1967. 10.1093/brain/awt106 23715094 PMC3673467

[B68] Westman J. C. (1976). Gesell and amatruda’s developmental diagnosis: the evaluation and management of normal and abnormal neuropsychologic development in infancy and early childhood. *Am. J. Psychiatry* 133:351. 10.1176/ajp.133.3.351

[B69] Wible B. Nicol T. Kraus N. (2005). Correlation between brainstem and cortical auditory processes in normal and language-impaired children. *Brain* 128 417–423. 10.1093/brain/awh367 15634732

[B70] Winer J. A. (2005). Decoding the auditory corticofugal systems. *Hear. Res.* 207 1–9. 10.1016/j.heares.2005.06.007 16091301

[B71] Yan J. Ehret G. (2002). Corticofugal modulation of midbrain sound processing in the house mouse. *Eur. J. Neurosci.* 16 119–128. 10.1046/j.1460-9568.2002.02046.x 12153536

