# Supplementary material for: Dysfunction of the Auditory Brainstem as a Neurophysiology Subtype of Autism Spectrum Disorder
Source: Front Neurosci. 2021 Mar 17;15:637079. doi: 10.3389/fnins.2021.637079 (PMC8010248; doi:10.3389/fnins.2021.637079)
Supplement: Supplementary Figure1 — Hypothesized model among cortex, subcortex, and language in ASD preschool children. [file Data_Sheet_1.PDF]

Table 1 Cluster of significant differences in cortical morphometry between TD and ASD

Measure	cluster	Size(mm2)	X	Y	Z	Number of vertices	Peak region
Thickness	1	1705.7	-26.3	23.8	-6	4290	left lateral orbitofrontal
	2	840.25	-7.3	37.5	13.5	1480	left rostral anterior cingulate
	3	726.89	-12.8	-11.1	67.7	1615	left superiorfrontal
	4	703.12	-38.1	50	-3.4	964	left rostral middlefrontal
	5	548.55	-4.1	-33.4	30.5	1271	left isthmus cingulate
	6	519.59	-36.8	-18.3	64.5	1128	left precentral
	7	1587.12	11	14.6	62.2	3337	right superiorfrontal
	8	1207.7	44	-12.8	20.7	3306	right postcentral
	9	1064.32	31	-41.1	-9	1793	right parahippocampal
	10	832.24	8.3	37	-3.9	1578	right rostral anterior cingulate
	11	799.48	14.3	-51.8	34.2	2096	right precuneus
	12	786.76	44	-67.7	7	1473	right inferiorparietal
	13	614.14	27.7	57.8	-9.5	925	right rostral middlefrontal
	14	483.62	61.5	-34.9	-14.2	773	right middletemporal
	15	459.16	39.1	-9.6	60.8	1044	right precentral
Area	1	1564.94	-33.9	-9.1	-29.1	2935	left fusiform
	2	814.35	-9.1	62.3	-7.5	1134	left frontalpole
	3	755.16	-50.2	6	5.7	1635	left parsopercularis
	4	2701.84	46.9	-21.1	-26.6	4687	right inferior temporal
Volume	1	651.25	44	-67.7	7	1110	right inferior parietal

Significant threshold was set at $p < 0.01$, Cluster corrected.
