# Supplementary material for: Dysfunction of the Auditory Brainstem as a Neurophysiology Subtype of Autism Spectrum Disorder
Source: Front Neurosci. 2021 Mar 17;15:637079. doi: 10.3389/fnins.2021.637079 (PMC8010248; doi:10.3389/fnins.2021.637079)
Supplement: Supplementary Table 1 — Cluster of significant differences in cortical morphometry between the TD group and ASD group. [file Data_Sheet_2.PDF]

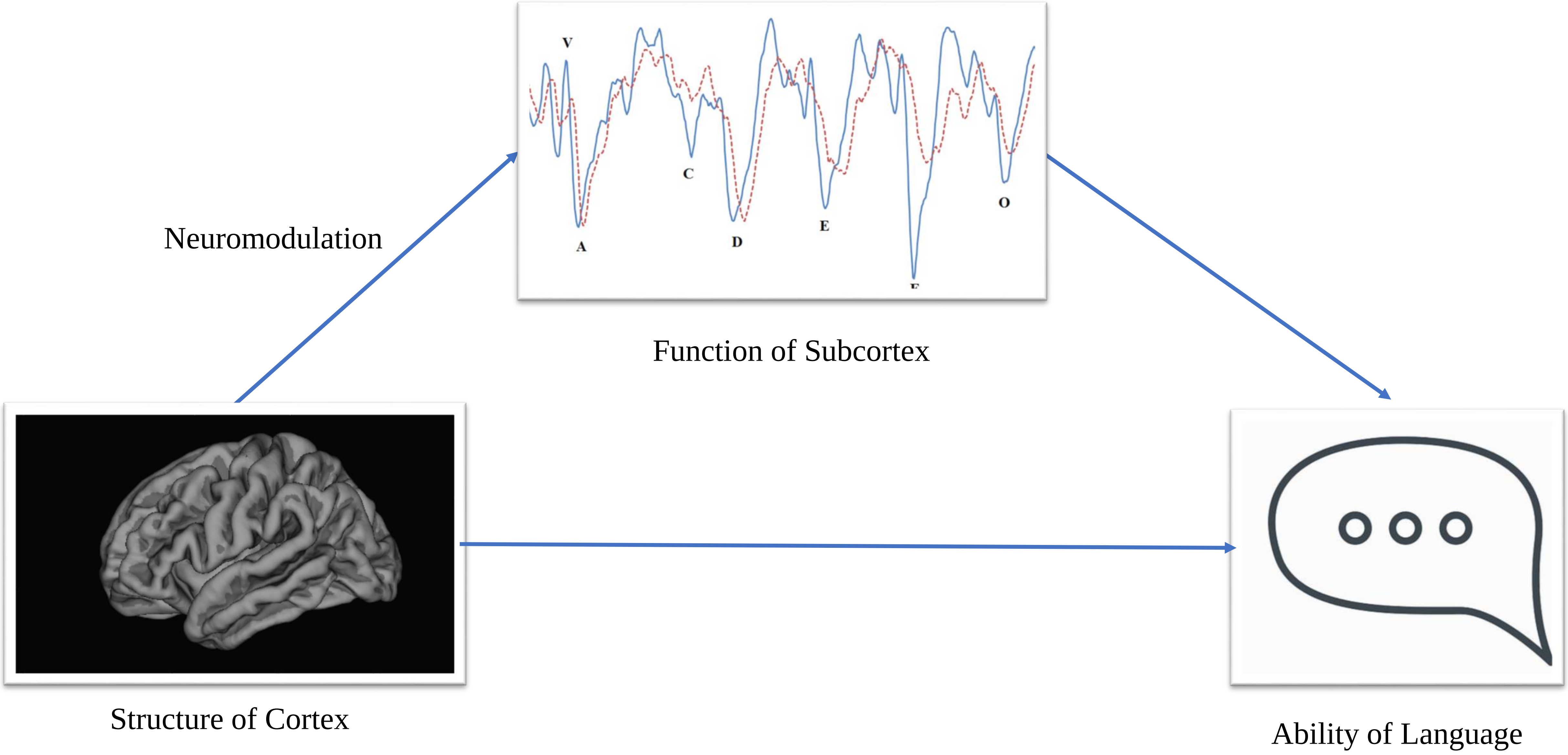

Figure 1 Hypothesized model among cortex, subcortex and language
